# Serological response to pandemic influenza A/H1N1 2009 and seasonal influenza A/H3N2 among health care workers (HCWs) in JIPMER, Puducherry

**DOI:** 10.1186/1471-2334-14-S3-P41

**Published:** 2014-05-27

**Authors:** G Nandhini, S Sujatha

**Affiliations:** 1 Department of Microbiology, JIPMER, Puducherry, India

## Background

Serum antibody titers against influenza viruses are regarded as markers of partial or complete immunoprotection. Antibody titer of ≥40 is associated with at least a 50% reduction in risk for infection or disease.

## Methods

Serum was collected from 138 HCWs including laboratory personnel, doctors and nurses working at JIPMER, Puducherry during August - October 2013. Details of influenza vaccination and laboratory confirmed influenza infection were noted. Hemagglutination inhibition assay was performed to determine the serum antibody levels against WHO reference antigens – pandemic influenza A (H1N1) A/California/07/2009, seasonal influenza A (H3N2) A/Victoria/361/2011.

## Results

Fifty of the HCWs had received pandemic influenza vaccination at least once in the previous three years. All HCWs had antibody titers ≥40 against seasonal influenza A/H3N2. Antibody titers against pandemic influenza A/H1N1 2009 ranged from ≤20 (16.7%) to 320 with 36.9% showing borderline protective titers (=40). Six of the vaccinated HCWs had non-protective antibody titers while 71 unvaccinated HCWs showed protective titers. Only one HCW developed seasonal influenza A/H3N2 infection despite having borderline protective antibody titer of 40 and his convalescent serum sample after two weeks showed fourfold rise in titers.

## Conclusion

This study showed protective antibody levels against pandemic influenza A/H1N1 2009 and seasonal influenza A/H3N2 among a large percentage of HCWs, regardless of vaccination status, which may be the primary reason for the low incidence of influenza cases encountered this year.

